# Targeted Therapy Recommendations for Therapy Refractory Solid Tumors—Data from the Real-World Precision Medicine Platform MONDTI

**DOI:** 10.3390/jpm10040188

**Published:** 2020-10-23

**Authors:** Hossein Taghizadeh, Matthias Unseld, Martina Spalt, Robert M. Mader, Leonhard Müllauer, Thorsten Fuereder, Markus Raderer, Maria Sibilia, Mir Alireza Hoda, Stefanie Aust, Stephan Polterauer, Wolfgang Lamm, Rupert Bartsch, Matthias Preusser, Kautzky-Willer A., Gerald W. Prager

**Affiliations:** 1 Department of Medicine I, Division of Clinical Oncology, Medical University of Vienna, 1090 Vienna, Austria; seyed.taghizadehwaghefi@meduniwien.ac.at (H.T.); matthias.unseld@meduniwien.ac.at (M.U.); martina.spalt@akhwien.at (M.S.); robert.mader@meduniwien.ac.at (R.M.M.); thorsten.fuereder@meduniwien.ac.at (T.F.); markus.raderer@meduniwien.ac.at (M.R.); wolfgang.lamm@meduniwien.ac.at (W.L.); rupert.bartsch@meduniwien.ac.at (R.B.); matthias.preusser@meduniwien.ac.at (M.P.); 2 Comprehensive Cancer Center Vienna, 1090 Vienna, Austria; leonhard.muellauer@meduniwien.ac.at (L.M.); maria.sibilia@meduniwien.ac.at (M.S.); mir.hoda@meduniwien.ac.at (M.A.H.); stefanie.aust@meduniwien.ac.at (S.A.); stephan.polterauer@meduniwien.ac.at (S.P.); 3 Clinical Institute of Pathology, Medical University Vienna, 1090 Vienna, Austria; 4 Department of Medicine I, Institute of Cancer Research, Medical University of Vienna, 1090 Vienna, Austria; 5 Department of Surgery, Institute of Cancer Research, Medical University of Vienna, 1090 Vienna, Austria; 6 Department of Obstetrics and Gynecology, Medical University of Vienna, 1090 Vienna, Austria; 7 Department of Medicine III, Division of Endocrinology and Metabolism, Medical University of Vienna, 1090 Vienna, Austria; alexandra.kautzky-willer@meduniwien.ac.at; 8 Department of Medicine III, Gender Medicine Unit, Medical University of Vienna, 1090 Vienna, Austria

**Keywords:** molecular profiling, immunohistochemistry, next-generation sequencing, precision medicine, targeted therapy, molecular oncology

## Abstract

Advanced therapy-refractory solid tumors bear a dismal prognosis and constitute a major challenge in offering effective treatment strategies. In this real-world retrospective analysis of our precision medicine platform MONDTI, we describe the molecular profile of 554 patients diagnosed with 17 different types of advanced solid tumors after failure of all standard treatment options. In 304 cases (54.9% of all patients), a molecular-driven targeted therapy approach could be recommended, with a recommendation rate above 50% in 12 tumor entities. The three highest rates for therapy recommendation per tumor classification were observed in urologic malignancies (90.0%), mesothelioma (78.6%), and male reproductive cancers (71.4%). Tumor type (*p* = 0.46), expression of p-mTOR (*p* = 0.011), expression of EGFR (*p* = 0.046), and expression of PD-L1 (*p* = 0.023) had a significant impact on the targeted therapy recommendation rate. Therapy recommendations were significantly more often issued for men (*p* = 0.015) due to gender-specific differences in the molecular profiles of patients with head and neck cancer and malignant mesothelioma. This analysis demonstrates that precision medicine was feasible and provided the basis for molecular-driven therapy recommendations in patients with advanced therapy refractory solid tumors.

## 1. Introduction

Many efforts were undertaken for a thorough and more profound understanding of cancer diseases to develop potent strategies in prevention, diagnosis, and therapy. Despite great scientific advances and major breakthroughs in cancer research, it still poses an enormous challenge to medicine.

Cancer-related mortality is the second leading cause of death worldwide after cardiovascular diseases, being responsible for around 1 in 6 deaths. In 2018, over 18 million people were diagnosed with cancer and over 9 million patients died of it. Thus, cancer globally constitutes a major health and socioeconomic challenge, accounting for roughly over 213 million disability-adjusted life years and with resulting annual costs of over USD 1 trillion to the global economy [1,2].

Currently, chemotherapeutic agents are still the mainstay in the therapy management of cancer.

In contrast to conventional systemic cytotoxic chemotherapy that inhibits DNA synthesis and mitosis and causes a broad range of significant treatment-adverse related events, targeted antitumoral agents—consisting mainly of antibodies and small molecular agents—interfere with and alter the signaling pathways of malignant cells to induce damage to the cancer cells.

In recent years, there has been an effort to develop targeted agents and thus to individualize and personalize therapy concepts in many cancer entities. This approach is known as precision medicine. The main rationale of precision medicine is to match a therapeutic agent to its corresponding molecular target, to allow a precise treatment tailored to a specific patient. It aims to achieve a better and more sustained response than more generic treatments, without damaging healthy cells and tissues.

Currently, in several cancer entities, tailored therapy attempts with immunotherapeutics or tyrosine kinase inhibitors are used, e.g., trastuzumab in HER2 positive breast cancer or gastric cancer [3,4]. Another important example is the combination of BRAF and MEK inhibition with dabrafenib and trametinib or vemurafenib and cobimetinib for the treatment of melanoma harboring a BRAF V600E mutation [5,6,7]. For the treatment of non-small cell lung cancer (NSCLC), molecularly targeted agents are already an integral part of therapeutic algorithms, including the inhibitors of the epidermal growth factor receptor (EGFR), including erlotinib, gefitinib, and osimertinib [8,9,10].

Recently, the FDA has also approved tissue-agnostic targeted drugs, including pembrolizumab for the treatment of microsatellite instability-high (MSI-H) tumors and larotrectinib and entrectinib for the therapeutic management of NTRK gene fusion-positive tumors.

Precision medicine is a rapidly evolving and highly dynamic field. Since 2010, several important large-scale prospective clinical trials have been conducted that herald the era of personalized medicine in the 21st century. These trials attempted to realize precision medicine in routine clinical practice and to eventually overcome the old habit to treat cancer entities with a “one size fits all” approach.

Several trials already demonstrated the clinical benefit of precision medicine by translating the concept of targeted therapies based on the molecular information of the cancer patients into longer overall survival (OS), higher overall response rate (ORR), and lower treatment-related adverse effects (TRAE) [11,12,13].

We conducted a single center retrospective cohort analysis of patients with 17 different types of advanced therapy refractory solid tumor that had been enrolled and profiled in our precision medicine platform MONDTI (molecular oncologic diagnostics and therapy) of the Medical University of Vienna. We sought to describe the potential, the likelihood, and the gender aspects of targeted therapy recommendations in patients with different types of advanced solid tumors without further standard treatment option.

## 2. Materials and Methods

### 2.1. Patients and Design of the Precision Medicine Platform

Patients with pretreated, advanced solid tumors who had progressed to all standard treatment options confirmed by response evaluation criteria in solid tumors 1.1 (RECIST 1.1) criteria were eligible for inclusion in our precision medicine platform, provided that tissue samples for molecular profiling were available. The specimens were either obtained by fresh tumor biopsy performed by physicians at the Department of Interventional Radiology or were provided by the archives of the Department of Pathology when tumor biopsy was not feasible. Patients had to have an Eastern Cooperative Oncology Group (ECOG) performance status of 0 or 1. Our precision medicine platform is not a clinical trial but intends to provide targeted therapy recommendations to patients where no standard anti-tumoral treatment is available. All patients in this analysis had to be at least 18 years old at the time of molecular analysis and had to provide informed consent before inclusion in our platform. This analysis was approved by the Institutional Ethics Committee of the Medical University of Vienna (Nr. 1039/2017).

In this single center, real-world, retrospective analysis of our precision cancer medicine platform MONDTI, we describe the molecular profile and the likelihood of targeted therapy recommendations for 554 patients diagnosed with 17 different types of advanced solid tumor, with at least 10 patients per tumor type. Tumor samples of the patients were examined using next-generation sequencing panels, immunohistochemistry, and fluorescence in situ hybridization, as described in detail below.

All profiles were reviewed by a multidisciplinary team for the evaluation of a targeted treatment recommendation in a molecular tumor board.

### 2.2. Tissue Samples

Formalin-fixed, paraffin-embedded tissue samples from patients with advanced solid tumors who had progressed to all standard therapy regimens were obtained from the archive of the Department of Pathology, Medical University of Vienna, Austria.

### 2.3. Cancer Gene Panel Sequencing

DNA was extracted from paraffin-embedded tissue blocks with a QIAamp Tissue KitTM (Qiagen, Hilden, Germany). In total, 10 ng DNA per tissue sample was provided for sequencing. The DNA library was created by multiplex polymerase chain reaction with the Ion AmpliSeq Cancer Hotspot Panel v2 (Thermo Fisher Scientific, Waltham, MA, USA) that covers mutation hotspots of 50 genes. The panel includes driver mutations, oncogenes, and tumor suppressor genes. By the middle of 2018, the gene panel was expanded using the 161-gene next-generation sequencing panel of Oncomine Comprehensive Assay v3 (Thermo Fisher Scientific, Waltham, MA, USA) that covers genetic alterations and gene fusions. All of the genes detected by the 50-gene panel and 87 genes detected by the 161-gene panel were hotspot alterations. See Supplementary Materials (Table S1) for a complete list of the gene panels. The Ampliseq cancer hotspot panel was sequenced with an Ion PGM (Thermo Fisher) and the Oncomine Comprehensive Assay v3 on an Ion S5 sequencer (Thermo Fisher Scientific, Waltham, MA, USA). The generated sequencing data were afterwards analyzed with the help of the Ion Reporter Software (Thermo Scientific Fisher). We referred to BRCA Exchange, ClinVar, COSMIC, dbSNP, OMIM, and 1000 genomes for variant calling and classification. The variants were classified according to a five-tier system comprising the modifiers pathogenic, likely pathogenic, uncertain significance, likely benign, or benign. This classification was based on the standards and guidelines for the interpretation of sequence variants of the American College of Medical Genetics and Genomics [14]. The variants pathogenic and likely pathogenic were taken into consideration for the recommendation of targeted therapy.

### 2.4. Immunohistochemistry

Immunohistochemistry (IHC) was performed using 2-μm-thin tissue sections read by a Ventana Benchmark Ultra stainer (Ventana Medical Systems, Tucson, AZ, USA). The following antibodies were applied: anaplastic lymphoma kinase (ALK) (clone 1A4; Zytomed, Berlin, Germany), CD20 (clone L26; Dako), CD30 (clone BerH2; Agilent Technologies, Vienna, Austria), DNA mismatch repair (MMR) proteins including MLH1 (clone M1, Ventana Medical Systems), PMS2 (clone EPR3947, Cell Marque, Rocklin, CA, USA), MSH2 (clone G219-1129, Cell Marque), and MSH6 (clone 44, Cell Marque), epidermal growth factor receptor (EGFR) (clone 3C6; Ventana), estrogen receptor (clone SP1; Ventana Medical Systems), human epidermal growth factor receptor 2 (HER2) (clone 4B5; Ventana Medical Systems), HER3 (clone SP71; Abcam, Cambridge, UK), C-kit receptor (KIT) (clone 9.7; Ventana Medical Systems), MET (clone SP44; Ventana), NTRK (clone EPR17341, Abcam), phosphorylated mammalian target of rapamycin (p-mTOR) (clone 49F9; Cell Signaling Technology, Danvers, MA, USA), platelet-derived growth factor alpha (PDGFRA) (rabbit polyclonal; Thermo Fisher Scientific), PDGFRB (clone 28E1, Cell Signaling Technology), programmed death-ligand 1 (PD-L1) (clone E1L3N; Cell Signaling Technology till mid-2018; as of mid-2018, the clone BSR90 from Nordic Biosite, Stockholm, Sweden is used), progesteron receptor (clone 1E2; Ventana), phosphatase and tensin homolog (PTEN) (clone Y184; Abcam), and ROS1 (clone D4D6; Cell Signaling Technology).

To assess the immunostaining intensity for the antigens EGFR, p-mTOR, PDGFRA, PDGFRB, and PTEN, a combinative semiquantitative score for immunohistochemistry was used. The immunostaining intensity was graded from 0 to 3 (0 = negative, 1 = weak, 2 = moderate, 3 = strong). To calculate the score, the intensity grade was multiplied by the percentage of corresponding positive cells: (maximum 300) = (% negative × 0) + (% weak × 1) + (% moderate × 2) + (% strong × 3).

The immunohistochemical staining intensity for HER2 was scored from 0 to 3+ (0 = negative, 1+ = negative, 2+ = positive, 3+ = positive) pursuant to the scoring guidelines of the Dako HercepTestR from the company Agilent Technologies (Agilent Technologies, Vienna, Austria). In the case of HER2 2+, a further test with HER2 in situ hybridization was performed to verify the HER2 gene amplification.

Estrogen receptor and progesterone receptor stainings were graded according to the Allred scoring system from 0 to 8. MET staining was scored from 0 to 3 (0 = negative, 1 = weak, 2 = moderate, 3 = strong) based on a paper by Koeppen et al. [15]. For PD-L1 protein expression, the tumor proportion score was calculated, which is the percentage of viable malignant cells showing membrane staining. In addition, as of 2019, the expression is also determined by the combined positive score.

The intensity of immunostaining intensity of a specific biomarker, including p-mTOR, HER2, PDGFR, PD-L1, is associated with the efficacy of the respective targeted therapy [16,17,18,19,20,21].

ALK, CD30, CD20, and ROS1 staining were classified as positive or negative based on the percentage of reactive tumor cells, however without graduation of the staining intensity. In ALK or ROS1 positive cases, the presence of a possible gene translocation was evaluated by fluorescence in situ hybridization (FISH).

All antibodies used in this study were validated and approved at the Clinical Institute of Pathology of the Medical University of Vienna and are used in routine IHC staining for clinical purposes. The antibodies have been validated—by proper positive and negative tissue controls and by non-IHC methods such as immunoblotting and flow cytometry—to detect the respective epitope of the antigens. For the control, the use of the antibodies was optimized in terms of intensity, concentration, signal/noise ratio, incubation time, and blocking. The negative control was conducted by omitting the primary antibody and by substitution of isotype-specific antibody and serum at the exact same dilution and laboratory conditions as the primary antibody to preclude unspecific binding.

For the positive control, the antibodies were shown not to cross-react with closely related molecules of the target epitope.

The status of MSI was analyzed by the MSI Analysis System, Version 1.1 (Promega Corporation, Madison, WI, USA).

### 2.5. Fluorescence In Situ Hybridization (FISH)

FISH was applied only in selected cases to verify PTEN loss. FISH was performed with 4-μm-thick formalin-fixed, paraffin-embedded tissue sections. The following FISH probe was utilized: PTEN (10q23.31)/Centromere 10 (ZytoVision, Bremerhaven, Germany). Two hundred cell nuclei per tumor were evaluated. The PTEN FISH was considered positive for PTEN gene loss with ≥30% of cells with only one or no PTEN signals. A chromosome 10 centromere FISH probe served as a control for ploidy of chromosome 10.

### 2.6. Multidisciplinary Team for Precision Medicine

After thorough examination of the molecular profile of each tumor sample by a qualified and competent molecular pathologist, the results and findings were reviewed in a multidisciplinary team (MDT) meeting that was held every other week.

Members of the MDT included molecular pathologists, radiologists, clinical oncologists, surgical oncologists, and basic scientists. The MDT recommended the targeted therapy based on the specific molecular profile of each patient. The targeted therapies included tyrosine kinase inhibitors, checkpoint inhibitors (e.g., anti- PD-L1 monoclonal antibodies), and growth factor receptor antibodies with or without endocrine therapy. The treatment recommendations by the MDT were prioritized dependent on the level of evidence from high to low according to phase III to phase I trials. Recommendations based on phase III, phase II, and phase I were designated as high, intermediate, and low, respectively.

In cases where more than one druggable molecular aberration was identified, the MDT recommended a therapy regimen to target as many molecular aberrations as possible, with special consideration of the toxicity profile of each antitumoral agent and their potential interactions. Since all patients were given all available standard treatment options for their cancer disease prior to their inclusion in our precision medicine platform, nearly all targeted agents were suggested as off-label use. If the tumor profile and the clinical characteristics of a patient met the requirements of a clinical trial for targeted therapies that was open for inclusion in our cancer center, patients were preferentially asked if they wanted to participate in the respective trial.

### 2.7. Study Design and Statistics

This study is a retrospective single center cohort analysis of 17 different types of advanced solid tumors, with at least 10 patients per tumor type. The objective was to describe the molecular portrait and to evaluate the likelihood and the molecular and gender aspects of a targeted therapy recommendation for common tumor types. Rare tumor types with less than 10 patients per tumor type discussed in our MONDTI platform over this seven-year period were excluded. We also used the method of frequency distribution to delineate the characteristics of the cancer patients. We used the method of frequency distribution to delineate the characteristics of the cancer patients.

Since our study had an exploratory and hypothesis-generating design, no adjustment for multiple testing was used [22]. Binary logistic regression analysis was employed to assess the influence of various factors on the therapy recommendation rate. To evaluate whether our dataset has a normal distribution, Shapiro–Wilk test and Kolmogorov–Smirnov test were utilized. To examine gender-specific differences, Chi-squared test χ2 and Mann–Whitney U test were applied.

For statistical analysis, the software package IBM SPSS Statistics Version 26 was used.

## 3. Results

From June 2013 to January 2020, 554 patients diagnosed with 17 different types of advanced therapy refractory solid tumors, with at least 10 patients per tumor entity, were included in this retrospective cohort analysis. This analysis is from the total cohort of our platform MONDTI, which has so far profiled 580 patients with various advanced cancer types. In this analysis, all patients were Caucasians. The median age at initial diagnosis was 54.3 years, ranging from 18 to 81 years, and the median age at the time when the molecular profiling was performed was 57.4 years, ranging from 18 to 84 years (Table 1). The tumor tissue was obtained from biopsy or during surgical intervention.

The five most frequent tumor types were gynecologic malignancy (*n* = 90; 16.1%), colorectal cancer (*n* = 56; 10.0%), tumor of the central nervous system (*n* = 55; 9.9%), squamous cell carcinoma of the head and neck (*n* = 44; 8.4%), and neuroendocrine carcinoma (*n* = 41; 7.4%), with details provided in Table 2.

At the time of molecular profiling, all patients had an advanced solid tumor which was refractory to therapy, all lines of standard treatment having been exhausted. Patients received between 1 and 5 lines of prior systemic chemotherapy; 287 patients had undergone a surgical intervention (51.8%).

In total, 397 tumor samples (71.7%) were tested with the 50-gene panel and 166 specimens (28.3%) were analyzed with the 161-gene panel.

**Figure 1:**
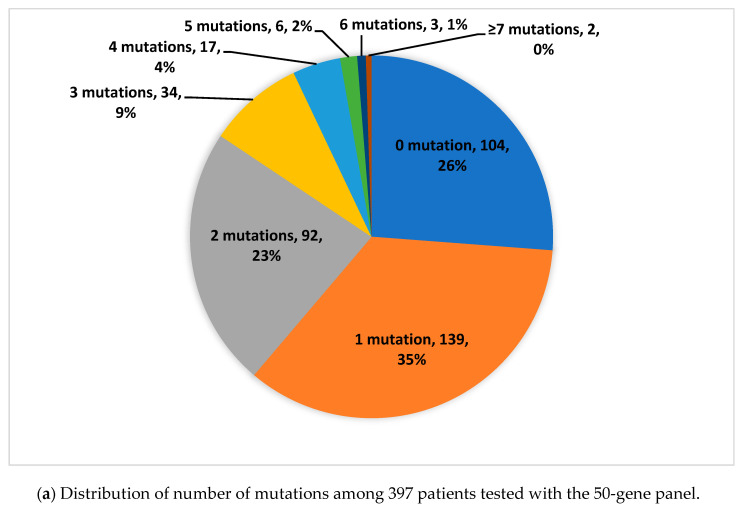

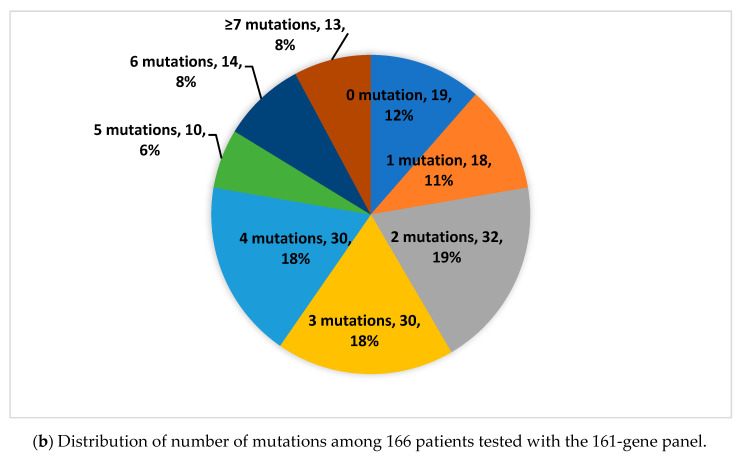
Distribution of number of mutations among the patients.

In total, we identified 1143 genomic aberrations in 441 (79.6%) patients: the 10 most frequent were TP53 (*n* = 228; 19.9%), KRAS (*n* = 103; 9.0%), PIK3CA (*n* = 54; 4.7%), PTEN (*n* = 35; 3.2%), APC (*n* = 28; 2.4%), CDKN2A (*n* = 28; 2.4%), NOTCH1 (*n* = 26; 2.3%), ATM (*n* = 25; 2.2%), SMAD4 (*n* = 19; 1.7%), IDH1 (*n* = 17, 1.5%). In 113 (20.4%) patients, no genetic alterations were detected. The inter- and intratumoral genomic profile was heterogeneous and mutations were seen in 123 different genes tested with the 161-gene panel (see Figure 1 and Table 3). The median number of mutations was two in the whole cohort. The median numbers of mutations were one and two when tested with the 50-gene panel and 161-gene panel, respectively.

**Figure 2:**
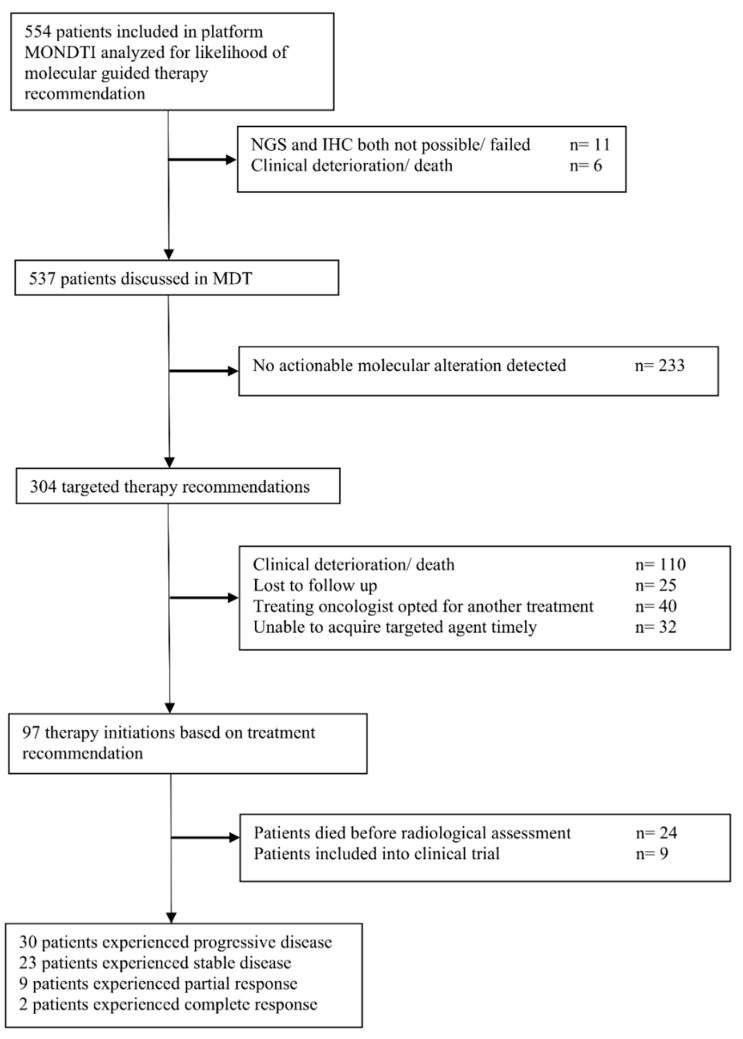
Flow of patients.

The next generation sequencing (NGS) analysis rate was high at 98.0%. Only in 11/554 (1.9%) patients, the NGS run failed. In 31/554 (5.6%) cases, IHC could not be performed (see Figure 2, which shows the flow of patients).

The studied population included 279 men and 275 women. The mutation rate was almost equal between the two genders: 48.9% in men versus 51.0% in women. The targeted recommendation rate, however, was slightly higher for men (53.6%, *n* = 163) when compared with women (46.4%, *n* = 141).

IHC revealed expression of p-mTOR (*n* = 419; 75.1%), EGFR (*n* = 386; 69.1%), PDGFRA (*n* = 183; 32.8%), PDGFRB (*n* = 45; 8.1%) MET (*n* = 178; 31.9 %), KIT (*n* = 35; 6.3%), HER2 (*n* = 36; 6.5%), HER3 (*n* = 58; 10.4%), PD-L1 (*n* = 92; 16.5%). In 57 cases (10.3%), loss of PTEN signal was reported. Seven patients (1.3%) had an MSI high status.

In total, we identified 33 gene fusions in our cohort (see Table 4).

In over half (*n* = 304, 54.9%) of the 554 patients, a targeted therapy was suggested, based on the identified molecular aberrations. The recommendation rate was over 50% in 12 different solid tumors. The five highest rates for therapy suggestion were observed in urologic malignancies (90.0%), mesothelioma (78.6%), male reproductive cancers (71.4%), tumors of the central nervous system (67.8%), and squamous cell carcinoma of the head and neck (SCCHN) (65.9%). In contrast, the three lowest rates were seen in breast cancer (38.1%), pancreatic ductal adenocarcinoma (31.6%), and diffuse large B-cell lymphoma (30.0%). We refer here to Table 2.

Of the 304 targeted treatment suggestions, 262 (86.2%) were mainly derived from the molecular information provided by IHC, while only in 39 cases (12.8%), the recommendation was mainly based on the genomic variations. In three cases (1%), the targeted therapy strategy was tailored based on the detection of FGFR fusion genes.

In total, 42 different antitumoral agents were recommended, either in combination or as a monotherapy. The three most frequently applied therapy regimens included the PD-1 inhibitors pembrolizumab and nivolumab (*n* = 62; 20.4%), the anti EGFR antibodies cetuximab and panitumumab (*n* = 29; 9.5%), and everolimus monotherapy (*n* = 26; 8.6%) (see Table 5).

The level of evidence was high, intermediate, and low in 25 (8.2%), 99 (32.6%), and 171 (56.3%) cases, respectively. Nine patients were enrolled in a clinical trial.

Eventually, 97 patients (17.5%) received the molecular guided treatment and thus experienced a change in clinical management because of the generated molecular information. Six out of 97 patients (6.2%) received on-label treatment. Nine of the 97 patients (9.3%) were treated in a clinical trial; 24 of 97 patients (24.7%) died before a radiological assessment could be performed; 30 patients (30.9%) did not respond and experienced a progressive disease. Stable disease was achieved in 23 patients (23.7%). Partial response and complete response were observed in nine (9.3%) and two (2.1%) patients, respectively. Thus, the disease control rate (DCR) was 35.1% and the overall response rate (ORR) was 11.3% in those patients who received the targeted therapy. Related to the whole cohort, the DCR was 6.1% (34/554) and the ORR was 2.0% (11/554).

The application of the Shapiro–Wilk test suggested that the distribution of age and genetic mutations was not normally distributed.

To detect possible gender-specific differences regarding the recommendation rate, we excluded gender-specific cancer diseases (breast cancers, gynecologic, and male reproductive malignancies) and used the Chi-squared test χ^2^. The test revealed a significant difference regarding the recommendation rate in the total cohort in favor of the male patients (*p* = 0.015). On the level of tumor subtypes, the Chi-squared test χ^2^ demonstrated a significant gender-specific difference in patients with SCCHN (*p* = 0.0027) and malignant mesothelioma (*p* = 0.008). Male patients with SCCHN had significantly more often PD-L1 expression than female patients (10/28 men versus 1/16 women; *p* = 0.030). Similarly, male patients with malignant mesothelioma had significantly more often PDGFRα expression than women (6/9 men versus 0/6 women; *p* = 0.017). After exclusion of these two tumor types, the gender-specific differences were not significant anymore (*p* = 0.24). These gender differences in the molecular profile of these two tumor entities are reflected by the type of targeted therapy recommendation.

In the next step, we investigated the effects of age, tumor type, and molecular profile on therapy recommendation using a binary logistic regression analysis, which showed that several of these factors had a significant impact on the recommendation rate: tumor type (*p* = 0.46), expression of *p*-mTOR (*p* = 0.011), expression of EGFR (*p* = 0.046), and expression of PD-L1 (*p* = 0.023).

Other parameters including age (*p* = 0.855), number of mutations (*p* = 0.850), expression of PDGFRα (*p* = 0.097), and expression of PDGFRβ (*p* = 0.420) were not significantly associated with therapy recommendation. The omnibus tests of model coefficients for the binary logistic regression were highly significant (*p* < 0.0001).

By using the Mann–Whitney U test, we could not find any gender-specific differences regarding age (*p* = 0.250) or number of mutations (*p* = 0.390). However, the Chi-squared test χ2 revealed, after exclusion of gender-specific cancer diseases, five different genetic mutations that are significantly more common in men than in women: CDKN2A (*p* = 0.04), CTNNB1 (*p* = 0.002), KIT (*p* = 0.0005), SLX4 (*p* = 0.034), and VHL (*p* = 0.046).

The median time interval between the failure of the last standard treatment line and the start of the molecularly targeted therapy was 63 days.

## 4. Discussion

This comprehensive analysis presents data from a real-world precision medicine platform.

The MONDTI platform for precision medicine is an open, tissue-agnostic and molecular-driven platform that seeks to provide targeted therapy strategies to patients based on the respective molecular profile. In our platform, we could offer tailored therapy concepts in over 50% of our patients, with 19 different advanced solid tumors with recommendation rates well above 70% in selected entities. Our study demonstrates that precision medicine is implementable into clinical routine. Considering the clinical outcome of targeted therapies in this retrospective analysis, the outcome was relatively poor.

Related to the whole cohort, the DCR was 6.1% (34/554) and the ORR was 2.0% (11/554). There are several reasons that might explain this poor outcome.

Firstly, we observed a median turnaround time of more than two months between the failure of the last standard treatment line and the start of the targeted therapy. In this time interval, over 100 patients experienced clinical deterioration or died before the start of the targeted therapy. Nearly a quarter of the patients who eventually received the targeted therapy died prior to radiological assessment. One reason for the poor outcome of molecular-driven treatment approaches in this study is the relatively long turnaround time, during which patients do not receive effective therapy. Even if the targeted therapy is applied, it may not have enough time for the targeted therapy to unfold its full antitumorigenic potential.

Thus, time is a highly critical factor in the therapeutic management of therapy refractory solid tumors. Moreover, we detected a broad variety of mutations highlighting the well-known tumoral heterogeneity in cancer diseases [23,24].

Based on our data, the likelihood for rational identification of molecular-based treatment concepts was above 50% for 12 different solid tumors. However, the majority of these recommendations (88.8%) were not based on a high level of evidence.

Hence, the poor clinical outcome may be partly related to the long turnaround time, the extreme tumor heterogeneity, and the low level of evidence for therapy recommendations.

Thus, it is clinically relevant to consider these factors, particularly in patients for whom no guideline-based treatment is available anymore.

Interestingly, we observed in our cohort gender-specific differences in the molecular profile and therapy recommendations of SCCHN and mesothelioma patients.

The binary logistic regression analysis revealed that the expression of p-mTOR, EGFR, and PD-L1 significantly influenced therapy recommendations. This finding is reflected in the most common types of recommended targeted therapy: pembrolizumab and nivolumab, the anti EGFR antibodies cetuximab and panitumumab, as well as everolimus in monotherapy and in combination therapies.

Genomic profiling was performed in 98.0% patients, which is higher than or comparable to the rate reported by NEXT-1 (95%), MOSCATO 01 (89%), IMPACT/COMPACT (87%), SAFIR01 (70%), and SHIVA (67%) [13,25,26,27,28]. We detected 1143 genetic alterations and observed gender-specific differences regarding the distribution of the aberrations.

This study has several limitations. First, we acknowledge that our analysis was retrospective. Although all patients with advanced solid tumors with no further standard treatment options were included in this platform, this study is biased to a certain degree, since we included only patients with available tumor specimens for molecular profiling and a good ECOG status between 0 and 1.

Additionally, we did not consider the generally known dynamic of spatial and temporal intratumoral heterogeneity. We recommended the targeted therapy based on a molecular profile from one biopsy and from one timepoint, which was not necessarily close to molecular profiling. To overcome these limitations in future, liquid biopsy might be an additional practicable tool to monitor the dynamic molecular landscape of patients to revise and adapt the targeted therapy accordingly at any given timepoint. Particularly, early signs of treatment resistance may help to direct our therapy decisions using serial liquid biopsies. By reducing the turnaround time via liquid biopsy and by accelerating the creation of a molecular profile, the potential targeted agent could more likely be applied before the performance status of the patients deteriorates or before the molecular landscape changes and makes the therapy ineffective. Liquid biopsy would also be an interesting option for patients unfit to undergo a biopsy [29].

Another limitation of this study is that the found distribution of the mutations may be confounded by the employment of two different gene panels (50-gene panel versus 161-gene panel).

There are several burning issues to be addressed in future clinical trials and translational research. The first is to harmonize procedures and introduce international standards regarding the applied methods and treatment decision-making strategies, e.g., a standardized method for PD-L1 staining and scoring. International cut-offs in immunohistochemistry should be introduced and adhered to in order to achieve comparable results in clinical trials.

Several clinical trials have demonstrated the clinical benefit of tissue-agnostic molecular-guided treatment concepts and strategies in advanced stages of solid tumors. It would be important and interesting to introduce precision medicine at earlier stages of cancer disease to evaluate the efficacy of this treatment strategy. For instance, I-SPY 2 platform trial tests personalized treatment concepts for the neoadjuvant treatment of locally advanced breast cancer [30].

This analysis demonstrates that precision medicine was feasible and provided the basis for molecular-driven therapy recommendations in patients with advanced therapy refractory solid tumors. Studies are ongoing to define the clinical benefit of this approach in the real-life setting. Although the concept of molecular-guided therapy strategies is a relatively new concept, it has the potential to inform, shape, and enrich the antitumoral therapeutic armamentarium.

## Figures and Tables

**Table 1:** Patient characteristics (N = 554).

Patient Characteristics	Number
Men	279
Women	275
Median age at initial diagnosis	54.3 (18–87)
Median age at molecular profiling	57.4 (18–89)
Caucasian	554
Types of advanced solid tumors	17
Prior lines of antitumoral therapy	1–5

**Table 2:** Number of patients and recommendation rate.

Type of Solid Tumor	Number of Patients	Number of Recommendations and Recommendation Rate; Evidence Level for Recommendation	Outcome of Patients Who Received the Targeted Therapy
Urologic malignancy	10	N = 9; 90.0%; intermediate: *n* = 7, low: *n* = 2	PD: *n* = 3
Mesothelioma	14	N = 11, 78.6%; intermediate: *n* = 5, low: *n* = 6	SD: *n* = 1; PD: *n* = 3; died prior to assessment: *n* = 1
Male reproductive cancer	14	N = 10; 71.4%; intermediate: *n* = 5, low: *n* = 5	PR: *n* = 2; PD: *n* = 1; died prior to assessment: *n* = 2
Tumor of the central nervous system	55	N = 37; 67.8%; low: *n* = 37	PR: *n* = 2; SD: *n* = 4; PD: *n* = 3; died prior to assessment: *n* = 2
Squamous cell carcinoma of the head and neck	44	N = 29; 65.9%; high: *n* = 9, intermediate: 8, low: *n* = 12	SD: *n* = 3; PD: *n* = 4; died prior to assessment: *n* = 3
Sarcoma	17	N = 11; 64.7%; intermediate: *n* = 2, low: *n* = 9	CR: *n* = 1
Gynecologic malignancy	90	N = 58; 64.4%; high: *n* = 4; intermediate: *n* = 39, low: 13	SD: *n* = 4; PD = 2; died prior to assessment: *n* = 5; trials: *n* = 2
Hepatocellular carcinoma	16	N = 9; 56.3%; high: *n* = 1, intermediate: *n* = 1, low: 7	SD: *n* = 4; PD: *n* = 1; died prior to assessment: *n* = 2
Colorectal cancer	56	N = 30; 53.6%; high: *n* = 10, intermediate: *n* = 11, low: 6	PR: *n* = 2; trials: *n* = 3; PD: *n* = 1; died prior to assessment: *n* = 2
Lung cancer (without small cell lung cancer)	15	N = 9; 52.9%; high: *n* = 1, intermediate: *n* = 3, low: *n* = 5	PD: *n* = 3
Biliary Tract cancer	37	N = 19; 51.4%; intermediate: *n* = 6, low: *n* = 10	PR: *n* = 2; PD: *n* = 2; trials: *n* = 3; died prior to assessment: *n* = 2
Cancer of unknown primary	35	N = 18; 51.4%; low: *n* = 18	SD: *n* = 3; PR: *n* = 1; CR: *n* = 1; PD: *n* = 2; died prior to assessment: *n* = 1
Esophagogastric cancer	21	N = 9; 42.9%; low: *n* = 8	SD: *n* = 1; trial: *n* = 1; died prior to assessment: *n* = 1
Neuroendocrine carcinoma	41	N = 16; 39.0%; intermediate: *n* = 5; low: 11	SD: *n* = 1, PD = 3
Breast cancer	21	N = 8; 38.1%; intermediate: *n* = 5, low: *n* = 3	PD: *n* = 1
Pancreatic cancer	38	N = 12; 31.6%; low: *n* = 12	SD: *n* = 1; died prior to assessment: *n* = 2
Diffuse large B-cell lymphoma	30	N = 9; 30.0%; intermediate: *n* = 2, low: *n* = 7	SD: *n* = 1; PD: *n* = 1; died prior to assessment: *n* = 1
Total	554	N = 304, 54.9%	

**Table 3:** Detected molecular alterations.

Genomic Alteration	Absolute Numbers	Frequency in %									
TP53	228	19.9%	MET	9	0.8%	VA65:C90HL	4	0.3%	RHOA	2	0.2%
KRAS	103	9.0%	PTCH1	9	0.8%	CCND1	3	0.3%	ROS1	2	0.2%
PIK3CA	54	4.7%	RAD50	9	0.8%	CDH1	3	0.3%	SF3B1	2	0.2%
PTEN	37	3.2%	AKT1	8	0.7%	DDR2	3	0.3%	SRC	2	0.2%
APC	28	2.4%	FGFR3	8	0.7%	ESR1	3	0.3%	TERT	2	0.2%
CDKN2A	28	2.4%	SMARCB1	8	0.7%	FGFR4	3	0.3%	RHOA	2	0.2%
NOTCH1	26	2.3%	BRCA1	7	0.6%	HRAS	3	0.3%	ROS1	2	0.2%
ATM	25	2.2%	IDH2	7	0.6%	MAP2K1	3	0.3%	SF3B1	2	0.2%
SMAD4	19	1.7%	MSH6	7	0.6%	MYCL	3	0.3%	AKT2	1	0.1%
IDH1	17	1.5%	PALB2	7	0.6%	NTRK1	3	0.3%	AR	1	0.1%
PIK3R1	17	1.5%	SMARCA4	7	0.6%	PDGFRA	3	0.3%	AXL	1	0.1%
CTNNB1	16	1.4%	TSC1	7	0.6%	RAD51B	3	0.3%	CBL	1	0.1%
BRCA2	15	1.3%	ALK	6	0.5%	RNF43	3	0.3%	CD274	1	0.1%
RB1	15	1.3%	BAP1	6	0.5%	CDK4	2	0.2%	CDK4	1	0.1%
EGFR	14	1.2%	FGFR2	6	0.5%	CCND2	2	0.2%	CHEK2	1	0.1%
FANCA	14	1.2%	NBN	6	0.5%	CDK2	2	0.2%	FANCI	1	0.1%
POLE	14	1.2%	NF2	6	0.5%	CHEK1	2	0.2%	IGF1R	1	0.1%
TSC2	14	1.2%	SMO	6	0.5%	ERBB3	2	0.2%	JAK1	1	0.1%
ATR	13	1.1%	CDK12	5	0.4%	EZH2	2	0.2%	JAK2	1	0.1%
BRAF	13	1.1%	ERBB4	5	0.4%	FANCD2	2	0.2%	MAPK1	1	0.1%
NF1	13	1.1%	FGFR1	5	0.4%	FLT3	2	0.2%	MCL1	1	0.1%
ARID1A	12	1.0%	MLH1	5	0.4%	GNAQ	2	0.2%	MDM2	1	0.1%
CREBBP	12	1.0%	PMS2	5	0.4%	JAK3	2	0.2%	MDM4	1	0.1%
KIT	12	1.0%	PTPN11	5	0.4%	MAF	2	0.2%	MSH	1	0.1%
FBXW7	11	1.0%	ABL1	4	0.3%	MAX	2	0.2%	NFE2L2	1	0.1%
RET	11	1.0%	ATRX	4	0.3%	MSH2	2	0.2%	NTRK3	1	0.1%
SLX4	11	1.0%	CCND3	4	0.3%	mTOR	2	0.2%	PPP2R1A	1	0.1%
STK11	11	1.0%	ERBB2	4	0.3%	MYCN	2	0.2%	RICTOR	1	0.1%
NOTCH2	10	0.9%	KDR	4	0.3%	NTRK2	2	0.2%	TET2	1	0.1%
NOTCH3	10	0.9%	MRE11A	4	0.3%	PDGFRB	2	0.2%	UTR3	1	0.1%
SETD2	10	0.9%	NRAS	4	0.3%	PIK3CB	2	0.2%	AKT2	1	0.1%
GNAS	9	0.8%	RAD51D	4	0.3%	RAD51C	2	0.2%			

**Table 4:** Detected gene fusions.

Tumor Entity	Number of Gene Fusions	Type of Gene Fusions
Colorectal cancer	7	FGFR3-TACC3 (*n* = 2)
		WHSC1L1-FGFR1
		PTPRK-RSPO3
		FNDC3B-PIK3CA
		SND1-BRAF
		EIF3E-RSPO2
Tumors of the central nervous system	6	EIF3E-RSPO2
		ESR1-CCDC170
		TPM3-NTRK1
		FGFR3-TACC3
		BRAF-MRPS33
		ESR1-CCDC170
Squamous cell carcinoma of the head and neck	6	TBL1XR1-PIK3CA
		MYB-NFIB
		EIF3E-RSPO2
		FNDC3B-PIK3CA
		EIF3E-RSPO2
		FNDC3B-PIK3CA
Hepatocellular carcinoma	5	EIF3E-RSPO2 (*n* = 2)
		DNAJB1-PRKACA (*n* = 3)
Gynecologic malignancies	3	TBL1XR1-PIK3CA (*n* = 2)
		EIF3E-RSPO2 (*n* = 2)
		ESR1-CCDC170
Lung cancer	3	PCNX-RAD51B
		EIF3E-RSPO2
		PTPRK-RSPO3
Pancreatic ductal adenocarcinoma	1	TBL1XR1-PIK3CA
Biliary tract cancer	1	FGFR2-OFD1
Sarcoma	1	EIF3E-RSPO2

**Table 5:** Recommended agents in monotherapy and in combination therapies.

Type of Targeted Agent	Number of Recommendations in Monotherapy	Biomarkers for Targeted Therapy Recommendation	Type of Targeted Agents	Number of Recommendations in Combination Therapies	Biomarkers for Targeted Therapy Recommendation
PD-1 Inhibitor	62	PD-L1 expression, MSI-H status	Everolimus + Exemestane	21	p-mTOR expression and PTEN loss; estrogen receptor
EGFR inhibitor(Cetuximab/Panitumuab)	29	EGFR expression and RAS wildtype	Everolimus + Cetuximab	6	p-mTOR expression and PTEN loss; EGFR expression and RAS wildtype
Everolimus	26	p-mTOR expression and PTEN loss	Everolimus + Sorafenib	1	p-mTOR expression and PTEN loss; estrogen receptor
Imatinib	19	ABL, KIT, PDGFR	Everolimus + Carboplatin	1	p-mTOR expression and PTEN loss; ATM, BRCA1, BRCA2, PALB2
Crizotinib	14	ALK, ROS1	Trastuzumab + Pertuzumab	5	HER2
Sunitinib	14	FLT3, KIT, PDGFR	Trametinib + Dabrafenib	5	BRAF V600E
Afatinib	12	EGFR, HER2, HER3	Cetuximab + Irinotecan	5	EGFR expression and RAS wildtype
Regorafenib	9	ABL, FGFR, PDGFR, KIT,	Cetuximab + Vemurafenib	3	EGFR expression and RAS wildtype; BRAF V600E
Palbociclib	8	CDK4, CDK6	Cetuximab + Temsirolimus	2	EGFR expression and RAS wildtype; p-mTOR expression and PTEN loss
Cabozantinib	5	KIT, FLT-3, AXL, RET, MET	Lapatinib + Trastuzumab	2	EGFR and HER2
Ponatinib	4	ABL, FLT3, KIT, PDGFR, RET	Sunitinib + Anastrozol	1	FLT3, KIT, PDGFR; estrogen receptor
Olaparib	4	BRCA1, BRCA2	Idelalisib + Rituximab	1	PIK3CA; CD20
Pazopanib	3	PDGFR, FGFR3	Alpelisib + Fulvestrant	1	PIK3CA; estrogen receptor
Erlotinib	3	EGFR	Olaparib + platinum-based chemotherapy	1	BRCA1, BRCA2; ATM, BRCA1, BRCA2, PALB2
Pemigatinib	3	FGFR2	Pembrolizumab + Bevacizumab	1	PD-L1 expression; VEGFA
Platinum based chemotherapy	2	ATM, BRCA1, BRCA2, PALB2	Imatinib + Everolimus	1	ABL, KIT, PDGFR; p-mTOR expression and PTEN loss
Enasidenib	2	IDH2	Imatinib + Letrozole	1	ABL, KIT, PDGFR; estrogen receptor
Fulvestrant	2	Estrogen receptor	Bevacizumab + Paclitaxel	1	VEGFA
Androgen receptor antagonists	2	Androgen receptor	Bevacizumab + Everolimus	1	VEGFA; p-mTOR expression and PTEN loss
Temsirolimus	2	p-mTOR expression and PTEN loss	Total	304	
Nintedanib	2	FLT3, FGFR, PDGFR			
Tamoxifen	2	Estrogen receptor			
Lapatinib	2	EGFR, HER2			
Idelalisib	1	PIK3CA, PIK3R1			
T-DM1	1	HER2			
Trametinib	1	BRAF V600E			
AKT inhibitor	1	AKT			
Foretinib	1	MET			
Capmatinib	1	MET exon 14 skipping			
Dasatinib	1	ABL KIT, PDGFR			
Alemtuzumab	1	CD52			
Brentuximab Vedotin	1	CD30			
Vismodegib	1	SMO			
Vemurafenib	1	BRAF V600E			
Exemestane	1	Estrogen receptor			
Bevacizumab	1	VEGFA			

## Supplementary Materials

The following are available online at https://www.mdpi.com/2075-4426/10/4/188/s1, Table S1: List of gene targets in Oncomine Comprehensive Assay v3 (Thermo Fisher Scientific, Waltham, MA, USA)—161 gene panel.
