# Supplementary material for: Targeted Therapy Recommendations for Therapy Refractory Solid Tumors—Data from the Real-World Precision Medicine Platform MONDTI
Source: J Pers Med. 2020 Oct 23;10(4):188. doi: 10.3390/jpm10040188 (PMC7712019; doi:10.3390/jpm10040188)
Supplement: Supplementary file 1 [file jpm-10-00188-s001.pdf]

List of gene targets in Oncomine Comprehensive Assay v3 (Thermo Fisher Scientific, Waltham, MA, USA) – 161 gene panel

Hotspot genes	Full-length genes	Copy number genes	Gene fusions (inter- and intragenic)
AKT1	ATM	AKT1	ALK
ALK	BAP1	AR	AXL
AR	BRCA1	CCND1	BRAF
ARAF	BRCA2	CCNE1	EGFR
BRAF	CDKN2A	CDK4	ERBB2
BTK	FBXW7	CDK6	ERG
CBL	MSH2	EGFR	ETV1
CDK4	NF1	ERBB2	ETV4
CHEK2	NF2	FGFR1	ETV5
CSF1R	NOTCH1	FGFR2	FGFR1
CTNNB1	PIK3R1	FGFR3	FGFR2
DDR2	PTCH1	FGFR4	FGFR3
EGFR	PTEN	FLT3	NTRK1
ERBB2	RB1	IGF1R	NTRK3
ERB83	SMARCB1	KIT	PDGFRA
ERBB4	STK11	KRAS	PPARG
ESR1	TP53	MDM2	RAF1
EZH2	TSC1	MDM4	RET
FGFR1	TSC2	MET	ROS1
FGFR2	ARID1A	MYC	AKT2
FGFR3	ATR	MYCL	AR
FLT3	ATRX	MYCN	BRCA1
FOXL2	CDK12	PDGFRA	BRCA2
GATA2	CDKN1B	PIK3CA	CDKN2A
GNA11	CDKN2B	PPARG	ERB84
GNAQ	CHEK1	TERT	ESR1
GNAS	CREBBP	AKT2	FGR
HNF1A	FANCA	AKT3	FLT3
HRAS	FANCD2	ALK	JAK2
IDH1	FANCI	AXL	KRAS
IDH2	MLH1	BRAF	MDM4
JAK1	MRE11A	CCND2	MET
JAK2	MSH6	CCND3	MYB
JAK3	NBN	CDK2	MYBL1
KDR	NOTCH2	CDKN2A	NF1
KIT	NOTCH3	CDKN2B	NOTCH1
KNSTRN	PALB2	ESR1	NOTCH4
KRAS	PMS2	FGF19	NRG1
MAGOH	POLE	FGF3	NTRK2
MAP2K1	RAD50	NTRK1	NUTM1
MAP2K2	RAD51	NTRK2	PDGFRB
MAPK1	RAD51B	NTRK3	PIK3CA
MAX	RAD51C	PDGFRB	PRKACA
MED12	RAD51D	PIK3CB	PRKACB
MET	RNF43	RICTOR	PTEN
MTOR	SETD2	TSC1	RAD51B
MYD88	SLX4	TSC2	RB1
NFE2L2	SMARCA4		RELA
NRAS			RSPO2
PDGFRA			RSPO3
PIK3CA			TERT
PPP2R1A			
PTPN11			
RAC1			
RAF1			
RET			
RHEB			
RHOA			
SF3B1			
SMO			
SPOP			
SRC			
STAT3			
U2AF1			
XPO1			
AKT2			
AKT3			
AXL			
CCND1			
CDK6			
ERCC2			
FGFR4			
H3F3A			
HIST1H3B			
MAP2K4			
MDM4			
MYC			
MYCN			
NTRK1			
NTRK2			
PDGFRB			
PIK3CB			
ROS1			
SMAD4			
TERT			
TOP1			

List of gene targets in the Ion AmpliSeq Cancer Hotspot Panel v2 (Thermo Fisher Scientific, Waltham, MA, USA) – 50 gene panel

Hotspot genes
ABL1
AKT1
ALK
APC
ATM
BRAF
CDH1
CDKN2A
CSF1R
CTNNB1
EGFR
ERBB2
ERBB4
EZH2
FBXW7
FGFR1
FGFR2
FGFR3
FLT3
GNA11
GNAS
GNAQ
HNF1A
HRAS
IDH1
JAK2
JAK3
IDH2
KDR
KIT
KRAS
MET
MLH1
MPL
NOTCH1
NPM1
NRAS
PDGFRA
PIK3CA
PTEN
PTPN11
RB1
RET
SMAD4
SMARCB1
SMO
SRC
STK11
TP53
VHL
